# Surgical management of multiple coronary artery to coronary sinus fistulas with giant left circumflex artery and multivalvular infective endocarditis

**DOI:** 10.1186/s13019-024-02657-3

**Published:** 2024-04-06

**Authors:** Sang Bin Shin, Won Kyoun Park, Jae Won Choi, Jong Pil Jung, Chang Ryul Park, Yong Jik Lee, Gwan Sic Kim

**Affiliations:** 1 Department of Thoracic and Cardiovascular Surgery, Asan Medical Center, University of Ulsan College of Medicine, Seoul, Republic of Korea; 2 Department of Thoracic and Cardiovascular Surgery, Ulsan University Hospital, University of Ulsan College of Medicine, 877, Bangeojinsunhwando-ro, Dong-gu, Ulsan, 44033 Republic of Korea

**Keywords:** Coronary sinus fistula, Giant left circumflex artery, Aneurysm, Infective endocarditis

## Abstract

Coronary artery fistula (CAF) is characterized as a congenital or acquired abnormal communication between a coronary artery and any of the four chambers of the heart (coronary–cameral fistula) or great vessels (coronary arteriovenous fistula) bypassing the capillaries within myocardium. CAF is a rare disease, challenging to diagnose and treat depending on the anatomical location and type of the fistula and accompanying diseases. This study aims to report a case with multiple coronary artery to coronary sinus (CS) fistulas with giant left circumflex artery and multivalvular infective endocarditis.

## Introduction

CAF denotes a direct connection between coronary arteries and the heart chambers or great vessels. The drain flow through the fistula triggers a steal effect, diminishing capillary flow and heightening the risk of concurrent cardiac diseases. While asymptomatic cases discovered incidentally may warrant observation, patients diagnosed with other cardiac conditions—such as chronic ischemic damage, heart failure, aneurysmal changes, valve disease, or infective endocarditis—could benefit from corrective surgical intervention during procedures.

### Case

**Fig. 1:**
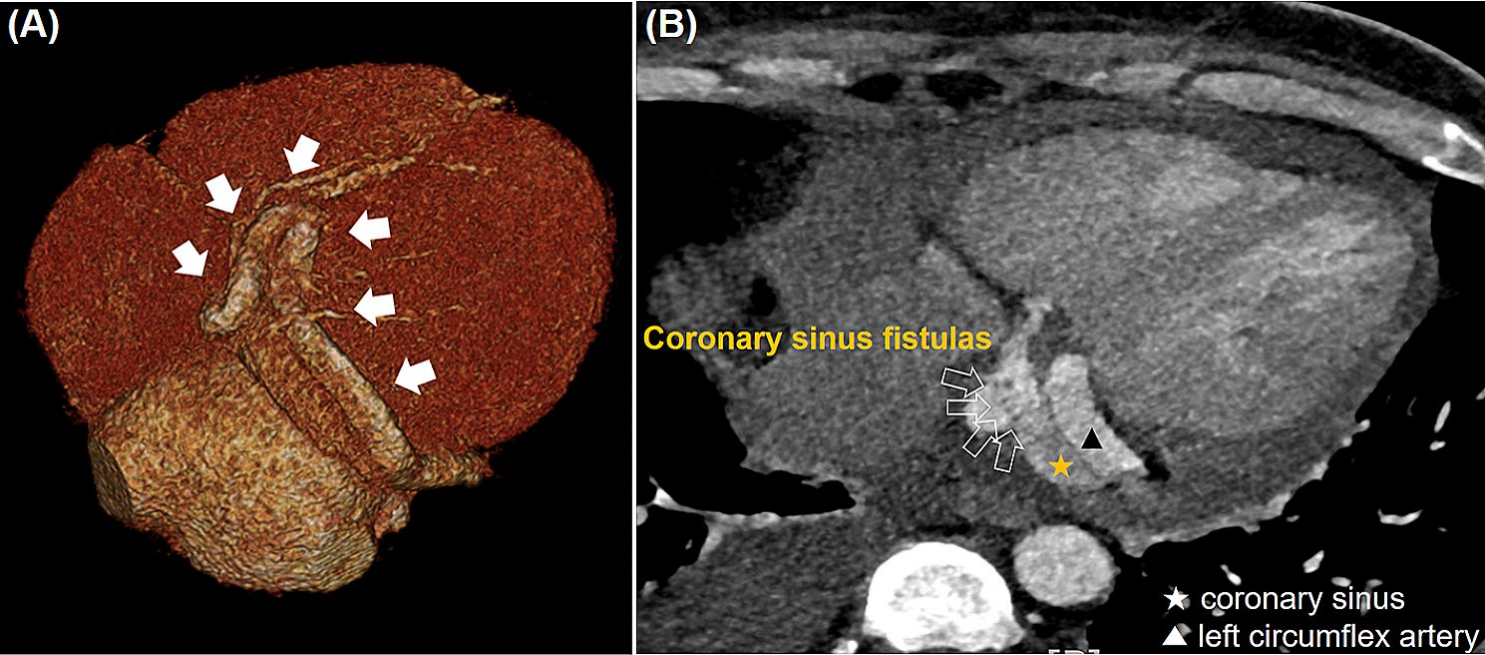
Cardiac computed tomography indicated that **(A)** LCx (white solid arrow) was enlarged to an overall diameter of approximately 1.2 cm while **(B)** multiple left circumflex CAFs (white blank arrow) draining into CS were identified

**Fig. 2:**
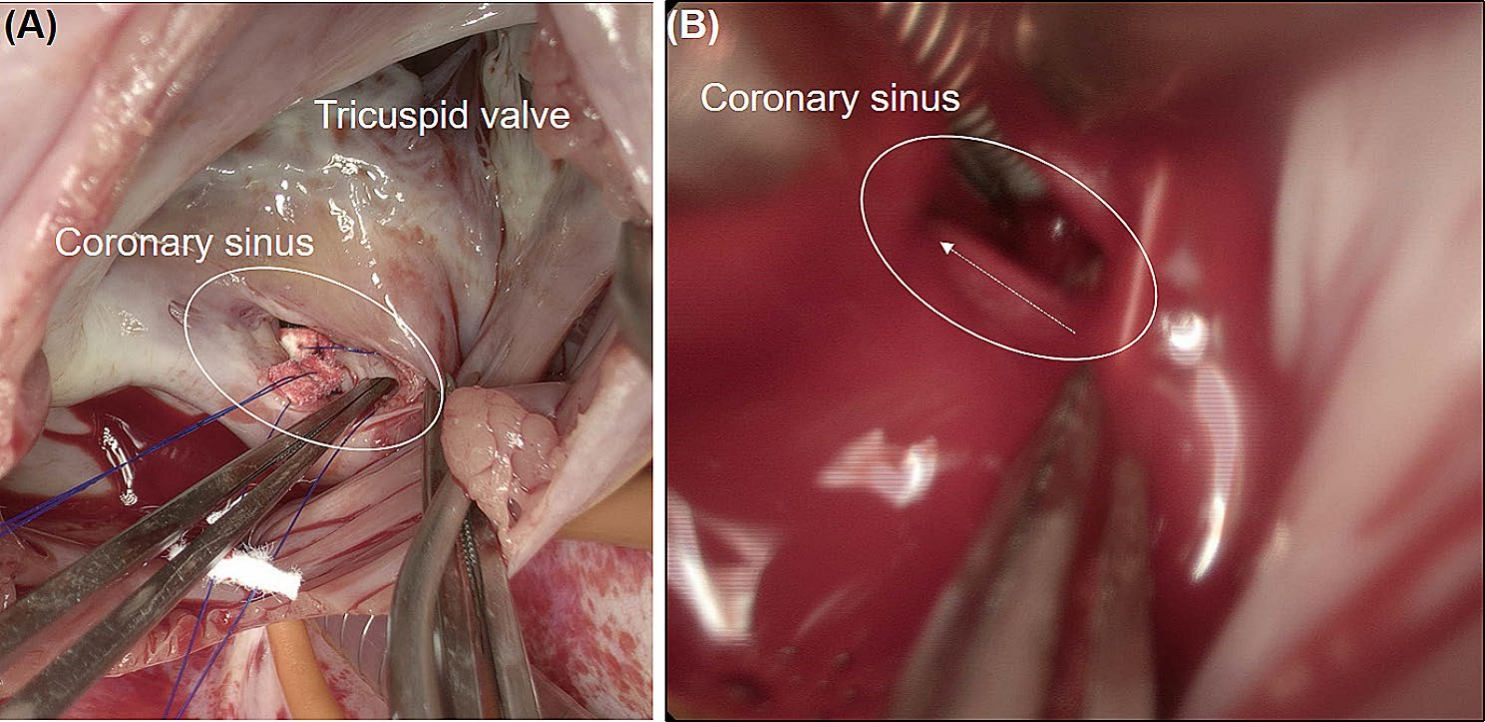
Intraoperative images; **(A)** direct suturing was performed using 4 − 0 prolene with pledget to close CS fistulas; **(B)** after de-airing and releasing aortic cross-clamp, it was checked whether any remaining shunt flow through fistula was observed; at this time, a strong shunt flow (white dotted arrow) was observed inside CS (white oval solid line)

**Fig. 3:**
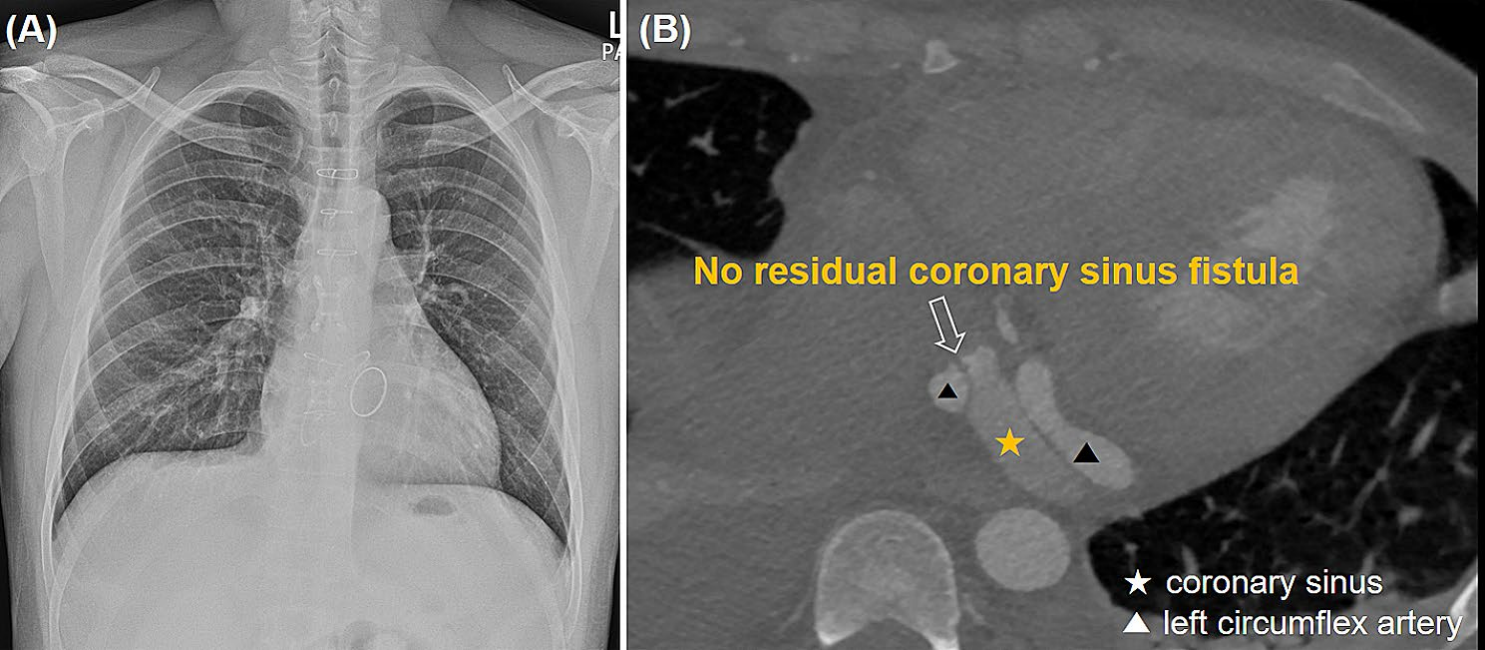
Postoperative images; **(A)** postoperative chest x-ray, **(B)** postoperative cardiac computed tomography indicated no residual (white blank arrow) fistula between LCx and CS

This study involves a 39-year-old male patient with no previous medical history who visited our hospital complaining of intermittent fever for 1 month. He had no history of recent overseas travel or invasive procedures, such as dental care or oriental acupuncture. The patient’s vital signs upon arrival were as follows: blood pressure 140/73 mmHg, regular heart rate 89 beats/min, respiratory rate 18 cycles/min, and body temperature 38.5℃. The laboratory test results indicated white blood cell count 8,410/mm^3^, hemoglobin 9.6 g/dl, total bilirubin 2.1 g/dl, C-reactive protein 4.59 mg/dl, and pro brain natriuretic peptide (pro BNP) 4,287 pg/mL. Conversely, other parameters were within normal limits. The chest radiographic findings showed a cardiothoracic (CT) ratio of 0.53, reflecting mild cardiomegaly. The electrocardiography indicated a normal sinus rhythm. The patient was initially admitted to the internal medicine department for evaluation of fever, and received empirical antibiotic treatment for 3 days before surgery. On the third day after admission, the transthoracic echocardiography (TTE) images indicated severe aortic regurgitation, severe mitral regurgitation, and moderate tricuspid regurgitation. Moreover, hyper-mobile vegetations of 3.0 × 1.2 cm^2^ and 1.2 × 0.7 cm^2^ in size were observed on aortic and mitral valves. The left and right ventricular sizes and systolic function were within normal limits. The report of the computerized tomography (CT) coronary angiogram exhibited that LCx was enlarged to an overall diameter of approximately 1.2 cm, and multiple left circumflex CAFs draining into CS were identified (Fig. 1). The patient was immediately referred to the cardiovascular and thoracic surgery department for surgery. On the account of the potential systemic embolic risk of hyper-mobile vegetations and progressing heart failure owing to multiple valve dysfunction, urgent surgery was performed with a diagnosis of multiple coronary sinus fistulas with giant left circumflex artery aneurysm and multivalvular infective endocarditis. The operation was conducted using conventional ascending aorta and bicaval bypass under moderate hypothermia. The retrograde infusion of histidine-tryptophan-ketoglutarate cardioplegic solution (Custodiol®) was utilized for myocardial protection. Vegetations were observed on aortic and mitral valve leaflets. Double valve replacement (St. Jude Medical Regent [23 and 29 mm in aortic and mitral positions]; St. Jude Medical Inc, St Paul, MN) was performed. After completing the valve replacements, the CS and tricuspid valve were examined through a right atriotomy. Four fistulas were visually confirmed inside the CS. Each fistula was approximately 1.5 mm in diameter. No vegetation on the tricuspid valve was observed, but significant central regurgitation was present. After direct suturing was implemented with pledgetted 4 − 0 Prolene to close the CS fistulas, de-airing maneuvers followed and the cross clamp was released. At that point we assessed whether any remaining shunt flow through the fistula was present. At this time, a strong shunt flow was observed inside CS (Fig. 2), because it was difficult to secure a surgical view due to the strong shunt flow just inside the coronary sinus, we first tried to secure a view by inserting a pump sucker into the coronary sinus to drain the blood. Nevertheless, because the visibility was poor, we were able to secure the view by blocking the entrance of the strong shunt flow with forcep. And additional direct suturing was performed, and additional direct suturing was performed. Moreover, tricuspid annuloplasty (TAP) was performed using 32 mm Edwards MC^3^ ring (Edwards LifeSciences, Irvine, CA), and the surgery was completed. The postoperative course was uneventful, and there were no complications. On the follow-up, the transthoracic echocardiogram and chest computed tomography did not demonstrate any abnormal findings (Fig. 3). The preoperative blood culture grew *Propionibacterium propionicum*. Empirical antibiotic treatment was initiated with ceftriaxone, doxycycline, and vancomycin. However, intraoperative tissue culture showed no growth. After a discussion with the Department of Infectious Diseases, doxycycline was discontinued, and intravenous antibiotics (ceftriaxone; 2 g one a day, vancomycin; 1 g one a day) were administered for 8 weeks. To assess the remnant shunt of CS fistula, first-pass radionuclide angiocardiography using technetium 99 m diethylamine triamine pentaacetic acid (TC99m-DTPA) was performed. The Qp/Qs ratio was measured at 1.2, indicating no residual shunt flow due to fistulas. The patient was discharged after switching to oral antibiotics (amoxicillin 1 g tid) and ≥ 2 weeks of medication. He did not complain of any symptoms or showed abnormal murmurs during the follow-up. No prosthetic valve dysfunction or recurrent abnormal flow were recorded were recorded at TTE over 18 months of follow up.

## Discussion

CAF is a rare disease, occurring in 0.002% general population and representing 0.4% cardiac malformations [1]. The origin of CAF is found in the right coronary artery, left anterior descending coronary artery, and LCx in 50–60%, 25–42%, and 18.8% of cases. The termination of the fistula is drained in this order: pulmonary artery, right ventricle, right atrium, and CS. Single fistulas are the most commonly observed, ranging from 74 to 90%, whereas multiple fistulas occur in 10.7–16% of all cases of CAFs [1, 2]. Thus, the present case (multiple LCx to CS fistulas) is a rare case among CAFs, considering the origin, termination, and number of fistulas.

Most CAFs are asymptomatic; however, when symptoms occur, the most commonly observed symptoms are exertional dyspnea, fatigue and angina. In addition, CAFs tend to grow larger with age and may be accompanied by various complications in older age. These complications primarily include the following:

1. Volume overload of the cardiac chamber can cause congestive heart failure, left ventricle hypertrophy, and arrhythmia.
2. Coronary dilation and aneurysmal changes due to shunt flow, and myocardial ischemia, may occur. When the diameter is four times larger than normal, or > 8 mm, it is classified as a giant aneurysm.
3. It may be accompanied by valvular dysfunction and infective endocarditis [1, 2].

In this case, the 39-year-old patient showed no previous major symptoms but giant aneurysmal changes in the LCx which was enlarged by > 8 mm and associated with infective endocarditis. Therefore, even if small CAFs and asymptomatic cases are detected, long-term follow-up is deemed necessary.

Diagnostic evaluation was performed by detecting anatomical abnormalities via TTE, transesophageal echocardiography, and computed tomography angiography. Moreover, to quantitatively evaluate the amount of left-to-right shunt, Qp/Qs ratio can be measured via first-pass radionuclide angiocardiography with 93% sensitivity and 97% specificity [3]. Although this test was not performed preoperatively due to emergency surgery, no significant remaining shunts were observed post-surgery.

CAF can be categorized into two types based on the connection method between origin and drain. “Side-to-side fistula” is proximal CAF. In this type, fistula connects to the coronary artery in the middle, resulting in dilation of the coronary artery at the proximal site of fistula opening. On the contrary, “End-to-side fistula” is distal CAF. Unlike proximal CAF, it is characterized by diffuse dilation, as the coronary artery’s endpoint forms the fistula while connecting to the drain site. The present case was diagnosed as an end-to-side fistula, which has diffuse dilatation.

Based on the 2009 ACC/AHA guidelines, for fistulas accompanied by symptoms, percutaneous or surgical closure should be indicated, which is Class I recommendation with Level of Evidence C [4]. Even if the indications for interventional treatment are not met, ongoing close monitoring is necessary due to the possibility of dilation and tortuous changes developing over time.

The surgical approach to treat fistulas depends on the anatomical conditions and type of fistula. If the fistula is exposed on epicardium, it can be divided and ligated or oversewn by making an arteriotomy. In cases where accessing fistula externally is challenging, an intracardiac approach can be applied [1]. The present case was diagnosed as “end-to-side” type, and an intracardiac approach was performed. After opening RA and confirming multiple fistulas in CS, direct suturing was attempted. If fistulas near CS are relatively confirmed, direct suturing is sufficiently possible. Moreover, it is possible to visually check whether any shunts remain immediately after releasing ACC, allowing additional suture to be taken. In this case, the surgery was able to be completed with additional sutures due to the presence of a strong remaining shunt after ACC was released. In this case, we report a rare case of multiple coronary artery to CS fistulas with giant left circumflex artery and multivalvular infective endocarditis.

## Data Availability

Data were obtained from health medical examination data from Ulsan University Hospital. Data sharing is not applicable.
